# A novel ZEB1/HAS2 positive feedback loop promotes EMT in breast cancer

**DOI:** 10.18632/oncotarget.14563

**Published:** 2017-01-09

**Authors:** Bogdan-Tiberius Preca, Karolina Bajdak, Kerstin Mock, Waltraut Lehmann, Vignesh Sundararajan, Peter Bronsert, Alexandra Matzge-Ogi, Véronique Orian-Rousseau, Simone Brabletz, Thomas Brabletz, Jochen Maurer, Marc P. Stemmler

**Affiliations:** ^1^ Department of General and Visceral Surgery, Medical Center, University of Freiburg, Faculty of Medicine, Freiburg, Germany; ^2^ German Cancer Consortium (DKTK), Heidelberg, Germany; ^3^ German Cancer Research Center (DKFZ), Heidelberg, Germany; ^4^ Institute for Surgical Pathology, Medical Center – University of Freiburg, Faculty of Medicine, Freiburg, Germany; ^5^ Tumorbank Comprehensive Cancer Center Freiburg, Medical Center, University of Freiburg, Faculty of Medicine, Freiburg, Germany; ^6^ Karlsruhe Institute of Technology, Institute of Toxicology and Genetics, Eggenstein-Leopoldshafen, Germany; ^7^ Amcure GmbH, Eggenstein-Leopoldshafen, Germany; ^8^ Department of Experimental Medicine I, Nikolaus-Fiebiger Center for Molecular Medicine, Friedrich-Alexander University of Erlangen-Nürnberg, Erlangen, Germany

**Keywords:** hyaluronic acid synthase 2 (HAS2), epithelial-mesenchymal transition, invasion, metastasis, CD44 signaling

## Abstract

Cancer metastasis is the main reason for poor patient survival. Tumor cells delaminate from the primary tumor by induction of epithelial-mesenchymal transition (EMT). EMT is mediated by key transcription factors, including ZEB1, activated by tumor cell interactions with stromal cells and the extracellular matrix (ECM). ZEB1-mediated EMT and motility is accompanied by substantial cell reprogramming and the acquisition of a stemness phenotype. However, understanding of the underlying mechanism is still incomplete. We identified hyaluronic acid (HA), one major ECM proteoglycan and enriched in mammary tumors, to support EMT and enhance *ZEB1* expression in cooperation with CD44s. In breast cancer cell lines HA is synthesized mainly by HAS2, which was already shown to be implicated in cancer progression. *ZEB1* and *HAS2* expression strongly correlates in various cancer entities and high *HAS2* levels associate with an early relapse. We identified HAS2, tumor cell-derived HA and ZEB1 to form a positive feedback loop as ZEB1, elevated by HA, directly activates *HAS2* expression. In an *in vitro* differentiation model HA-conditioned medium of breast cancer cells is enhancing osteoclast formation, an indicator of tumor cell-induced osteolysis that facilitates formation of bone metastasis. In combination with the previously identified ZEB1/ESRP1/CD44s feedback loop, we found a novel autocrine mechanism how ZEB1 is accelerating EMT.

## INTRODUCTION

Breast cancer is the most frequently diagnosed cancer in women with over 1.7 Million new cases and more than 500,000 patients succumbing to the disease every year. While the primary tumor is often detected and removed by surgery, 20–40% of patients suffer from tumor relapse due to cancer-cell spreading. These metastases that are frequently found in bones, lung, liver and the brain are the major cause for cancer-related deaths today [1, 2]. One critical event during cancer progression is the acquisition of a plastic, mesenchymal and motile phenotype by tumor cells originating from epithelial tissue. This allows tumor cells to delaminate from the primary tumor, break through the basement membrane, invade the surrounding tissues and eventually enter the blood stream. Upon transport to distant sites they extravasate from blood vessels and form micrometastases [3, 4]. This metastatic cascade requires the activation of embryonic programs: during cell spreading the epithelial-mesenchymal transition (EMT) is activated, followed by the induction of the reverse process, the mesenchymal-epithelial transition (MET), to allow colonization. The resulting metastases often resemble the primary tumor in grading and marker gene expression [1, 4, 5]. Molecularly, EMT is induced by the action of specific transcription factors of the ZEB (ZEB1/2), Snail (SNAI1/2) and basic helix loop helix families (TWIST1) [6–8]. It was shown that ZEB1 is a major driver of EMT, tumorigenesis and metastasis formation. It provides stemness properties, resistance to chemotherapy and its expression correlates with poor prognosis [9–13]. ZEB1 acts mainly as transcriptional repressor, regulating genes involved in cell adhesion, cell polarity and tight junctions as well as key epithelial microRNAs. In particular the miR-200 family that induces and stabilizes epithelial differentiation is embedded in a double negative regulatory feedback loop with ZEB1 [12, 14–16]. Moreover, ZEB1 induces epigenetic changes and cooperates with the Hippo-transducer YAP1 to also act as transcriptional activator of specific target genes involved in stemness, invasion and metastasis [9, 17, 18]. We recently found that ZEB1 also regulates differential splicing of the stem cell marker CD44. By repressing *ESRP1* epithelial-specific CD44v isoforms are switched to the standard isoform CD44s that further enhances *ZEB1* expression to maintain an EMT phenotype even in absence of external EMT stimuli [19].

Although these findings in part explain the molecular downstream function of ZEB1 within the tumor cell, efficient invasion and metastasis require interaction with the extracellular matrix (ECM) and the surrounding stroma as well. It is well known that tumor cells influence ECM composition to facilitate migration and invasion into the surrounding tissues [20, 21]. Hylaruronan (hyaluronic acid, HA) is one ubiquitously expressed simple proteoglycan that is present in the ECM. It is required for proper embryogenesis and regeneration, but often becomes deregulated in disease [20]. HA forms scaffolds for ECM assembly, functions as hydrogel to complex water molecules and directly signals to cells by interacting with a variety of cell surface receptors, including CD44 [20, 22]. HA is synthesized in different chain lengths differing in molecular weight and molecular function [23]. It was demonstrated that HA molecular weight composition is altered during tumorigenesis and that this alteration plays a major role in tumor progression [24, 25]. The tumor and metastasis promoting function is mediated in part by HA binding to and subsequent activation of CD44 [26, 27]. Autocrine and paracrine signals instruct tumor and stroma cells to deposit HA into the ECM, synthesized by three hyaluronic acid synthases (HAS1-3) [28]. HAS2 was shown to play a crucial role in the context of tumorigenesis. Elevated *HAS2* expression was correlated with an EMT phenotype in over 70% of metaplastic breast carcinoma [29]. Recently, it was shown that excess of HA generated by a *HAS2* transgene in a mouse model for breast cancer, accelerated the development of carcinoma [30].

Here we analyzed whether tumor cell secreted HA and *HAS2* expression is promoting ZEB1-dependent EMT and found that HA in combination with CD44s activates *ZEB1* expression. ZEB1 promotes additional HA synthesis by activation of *HAS2*, thereby generating an additional self-enforcing feedback loop involving HA/CD44s, ZEB1 and HAS2.

## RESULTS

### Extracellular hyaluronic acid triggers *ZEB1* expression

EMT and malignancy are ultimately connected with ECM reconstruction. Deposition of excess HA plays an important pro-invasive and pro-metastatic role [31]. We aimed to dissect how increased extracellular HA contributes to ZEB1-driven EMT and how its synthesis and secretion is regulated during tumor progression.

**Figure 1:**
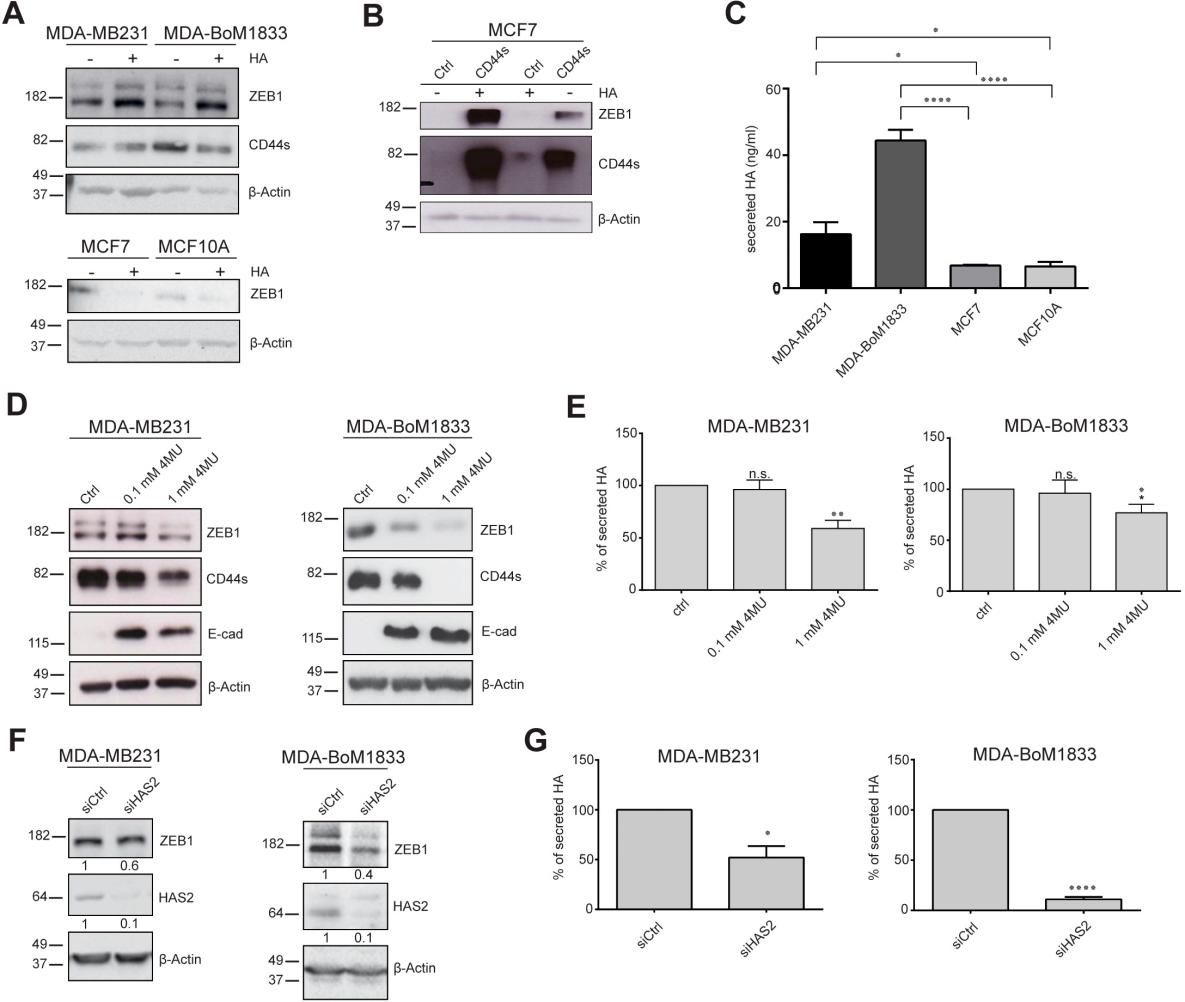
Hyaluronic acid (HA) is activating ZEB1 and CD44s expression (**A**) Western blot of mesenchymal and epithelial breast cancer cell lines showing increasing ZEB1 levels upon HA treatment in MDA-MB231 and MDA-BoM1833 whereas well-differentiated MCF7 and non-tumorigenic MCF10A cells are not affected. (**B**) ZEB1 protein levels in Western blots are increased upon combined CD44s transfection and HA treatment of MCF7 cells. Exogenous CD44s is stabilized and increased by HA treatment. (**C**) Measurement of secreted HA levels in breast cancer cell lines reveal increased levels in cells with a mesenchymal phenotype. (**D**) Blocking of HA production by 4-MU is inducing MET in MDA-MB231 and MDA-BoM1833 cells evident by decreasing ZEB1 and CD44s levels and activation of E-cad in Western blot (**E**) Quantification of secreted HA upon 4-MU treatment. (**F**) Western blot of MDA-MB231 and MDA-BoM1833 cells upon knockdown of *HAS2* shows ZEB1 downregulation, verified by quantification as indicated by numbers below individual blots. (**G**) Quantification of secreted HA in MDA-MB231 and MDA-BoM1833 upon *HAS2* knockdown reveals that HAS2 is the main enzyme for HA synthesis.

We made use of the triple-negative breast cancer cell line MDA-MB231 and its descendent line MDA-BoM1833, which has been selected for increased capacity to form bone metastasis upon injection of the parental cell line in mice [32]. Treatment of these two mesenchymal-like malignant cell lines with HA induced an increase in ZEB1 protein levels (Figure 1A). This 24-h short term treatment did not result in ZEB1-dependent CD44s accumulation yet. In contrast, addition of HA to the epithelial and noninvasive cancer cell line MCF7 and the mammary fibrocystic cell line MCF10A had rather opposite effects leading to further reduction of the already low levels of ZEB1, likely owing to the fact that one important receptor of HA, CD44s, is not expressed in MCF7 and MCF10A (Figure 1A) [19]. In line with this, overexpression of *CD44s* and treatment with extracellular HA showed a very robust upregulation of ZEB1 in MCF7 cells (Figure 1B). Hence, HA supports ZEB1-driven EMT that is enhanced by CD44s.

### HAS2 in breast cancer cell lines is crucial for autocrine HA-dependent activation of *ZEB1*

To further investigate this finding we interfered with autocrine HA synthesis by 4-methylumbelliferone (4-MU) treatment [33, 34]. MDA-MB231 and MDA-BoM1833 cells showed increased levels of HA secretion in comparison to the epithelial cell lines MCF7 and MCF10A (Figure 1C). Blocking HA synthesis in MDA-MB231 and MDA-BoM1833 resulted in robust downregulation of ZEB1 that coincided with reduction in CD44s and upregulation of E-cadherin (E-cad) on protein and mRNA levels, indicating activation of EMT (Figure 1D and 1E; Supplementary Figure S1A). Interestingly, 4-MU treatment also induced downregulation of total CD44 levels in contrast to a *ZEB1* knockdown that induced CD44 isoform switching as shown previously (Supplementary Figure S1A) [19].

HA is synthesized by three different hyaluronic acid synthases (HAS) encoded by *HAS1-3*, with different properties concerning the molecular size and function of the generated HA. In tumors HA is synthesized and secreted by stromal as well as by cancer cells. As *HAS2* expression and HAS2-generated HA have been shown to promote tumorigenesis, we asked whether HAS2 activity induces *ZEB1* expression in cancer cells. We used siRNA-mediated gene silencing that resulted in efficient *HAS2* knockdown and reduced the amount of secreted HA to 50% and 10% in MDA-MB231 and MDA-BoM1833 cells, respectively (Figure 1F and 1G; Supplementary Figure S1B). This resulted in a slight downregulation of ZEB1 protein levels only in MDA-BoM1833 cells, whereas transcripts were reduced to 50% and 40% in MDA-MB231 and MDA-BoM1833 cells, respectively (Figure 1F and Supplementary Figure S1B). Similar to the treatment with 4-MU, knockdown of *HAS2* led to a reduction of *CD44s*-specific transcripts below 60% in both cell lines. Interestingly, the effect of *HAS2* knockdown was not further enhanced by simultaneous depletion of all three HAS genes. Synthesis and secretion of HA was not blocked more efficiently, confirming that the majority of HA is produced by HAS2 whereas HAS1 and HAS3 play only minor roles in this context (Figure 1G and Supplementary Figure S1C). Taken together, these results show that secreted HA plays a key role in regulating *ZEB1* in an autocrine manner. Specifically the enzymatic activity of HAS2 is producing HA and promotes ZEB1-induction.

### *HAS2* strongly correlates with *ZEB1* expression in tumors and poor prognosis

**Figure 2:**
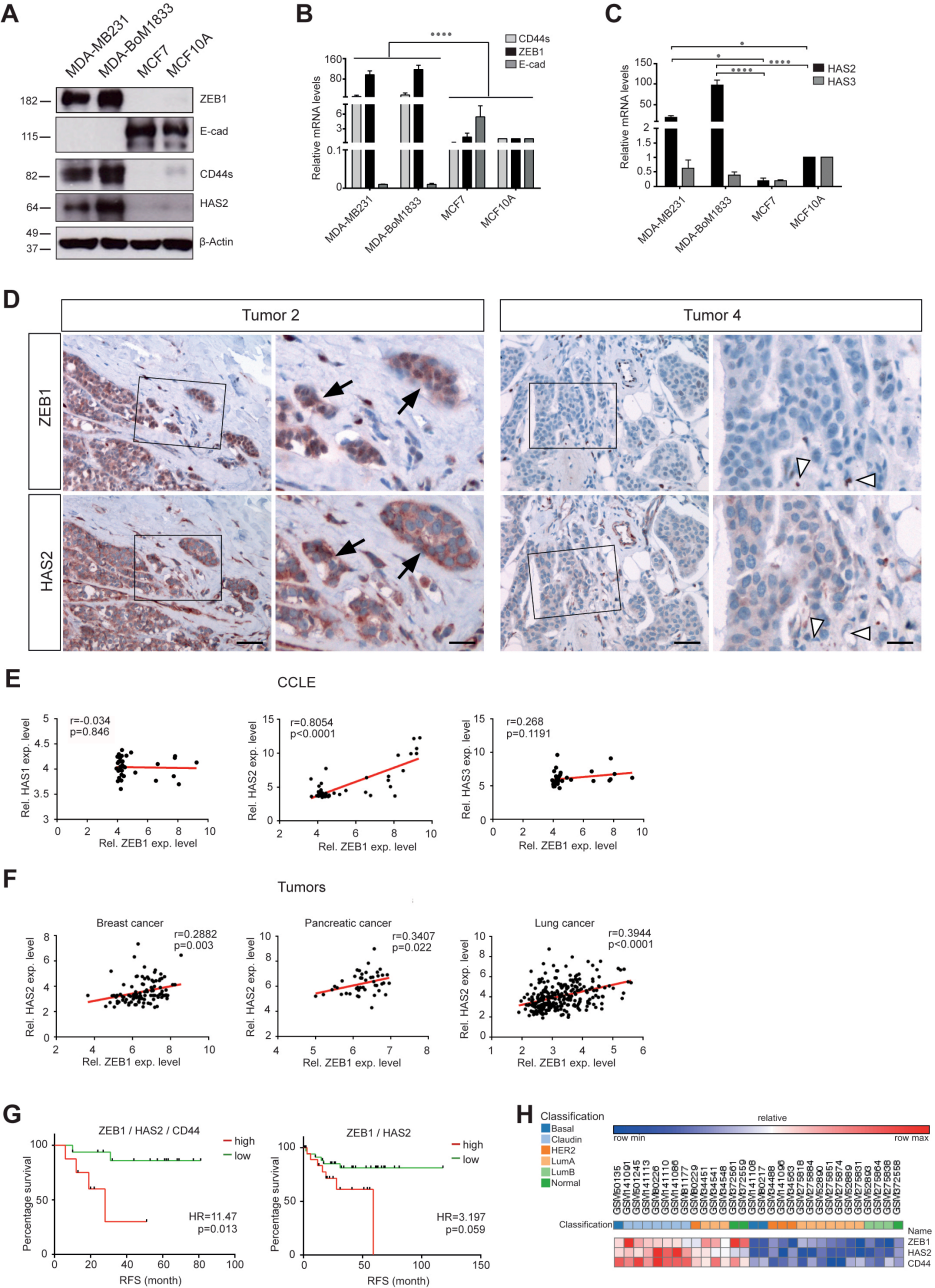
*HAS2* correlates with *ZEB1* expression and early relapse in breast cancer (**A**) Western blot of MDA-MB231, MDA-BoM1833, MCF7 and MCF10A cell lines. HAS2, CD44s and ZEB1 are coexpressed in mesenchymal-like cancer cells, whereas inversely only E-cad is expressed in the epithelial cell lines. (**B**, **C**) mRNA analysis of *CD44s*, *ZEB1* and *E-cad* (B) and *HAS2* and *HAS3* (C) in the four cell lines. (**D**) Immunohistochemical staining for ZEB1 and HAS2 on paraffin sections of breast cancer specimen. In ZEB1-positive tumors, areas of tumor cells that express ZEB1 are also positive for HAS2 (Tumor 2, arrows). In tumors without ZEB1 expression, tumor cells show absent or weak expression of HAS2 (Tumor 4). ZEB1-positive stroma cells (open arrowheads) are either HAS2 positive or negative. Scale bars, 50 μm (left panel), 20 μm (right panel). (**E**) Correlation analysis of microarray expression data from breast cancer cell lines (‘CCLE panel’, GSE36133). *ZEB1* correlates with *HAS2*, but not with *HAS1* and *HAS3* expression. (**F**) Correlation analysis of microarray expression data from tumor patients. *ZEB1* levels are correlated with *HAS2* in breast cancer (GSE42568), but also in pancreas (GSE28735) and lung cancer specimens (GSE41271). Pearson correlation coefficients r and *p*-values were computed and are indicated. (**G**) Kaplan-Meier plots of relapse-free (RFS) survival of upper 58%ile and lower 42%ile of combined high and low expression of *HAS2, CD44* and *ZEB1* (left) and of *HAS2* and *ZEB1* (right), derived from a microarray and follow-up study collection of 337 tumor samples (GSE18229). Increased *ZEB1*/*HAS2*/*CD44* and to lesser extend increased *ZEB1*/*HAS2* levels are correlated with early relapse. Hazard ratios (HR) and logrank *p*-values are given. (**H**) Heat map of expression of *ZEB1*, *HAS2* and *CD44* in patient samples in (G).

We wanted to understand whether EMT marker expression and *HAS2* were correlated in cell lines and tumor samples. MDA-MB231 and MDA-BoM1833 cells with a mesenchymal phenotype showed low expression of *E-cad*, whereas *ZEB1* and *CD44s* were highly expressed (Figure 2A and 2B). In line with increased HA secretion, *HAS2* was detected at substantial levels in these cell lines by Western blotting, immunofluorescence labeling and qRT-PCR analysis (Figure 2A and 2C, Supplementary Figure S2A). In contrast, the epithelial breast cancer and fibrotic cell lines MCF7 and MCF10A showed high *E-cad* levels and low levels of *ZEB1*, *CD44s* and *HAS2*, reflecting a weak or absent EMT signature (Figure 2A–2C, Supplementary Figure S2A). *HAS3* expression was indifferent in all cell lines and *HAS1* transcripts were not detectable (Figure 2C, data not shown). In breast cancer tissue sections we found robust co-expression of ZEB1 and HAS2 in tumor cell areas, whereas tumor cells missing ZEB1 were also negative for HAS2 (Figure 2D, Supplementary Figure S2B). This was also reflected by a correlation analysis of *ZEB1* and *HAS2* expression in microarray data sets of the CCLE (GSE3613332) [35] and the ‘NCI60’ (GSE58463) [36] panels (Figure 2E and Supplementary Figure S3). Moreover, genome-wide transcript analysis of tumor samples confirmed a close correlation between *ZEB1* and *HAS2* (but not with *HAS1* and *HAS3*) in breast, pancreas and lung cancer specimens (Figure 2F) (GSE42568 [37], GSE28735 [38] and GSE41271 [39]). Strikingly, high and low *HAS2* expression levels were associated with differences in the period to relapse (Figure 2G and Supplementary Figure S4). High *HAS2* levels alone already showed correlation with poor prognosis (Supplementary Figure S4C). Moreover, elevated levels of a *HAS2*, *CD44* and *ZEB1* geneset increased the hazard ratio from 2.84 to 11.47 in relapse-free and from 3.147 to 22.78 in overall survival studies (Figure 2G, Supplementary Figure S4A and S4C). Interestingly, the aggressive claudin-low subtype of triple-negative breast cancers showed high expression of *HAS2*, *CD44* and *ZEB1* (Figure 2H and Supplementary Figure S4D). *HAS1* and *HAS3* showed no correlation to survival (Supplementary Figure S4B and S4C). These results indicate that *HAS2*, *CD44* and *ZEB1* expression is correlated in cell lines and tumor samples. HAS2 is involved in tumor progression and acts in concert with ZEB1-driven EMT.

### HAS2 is necessary during TGFβ-induced EMT

**Figure 3:**
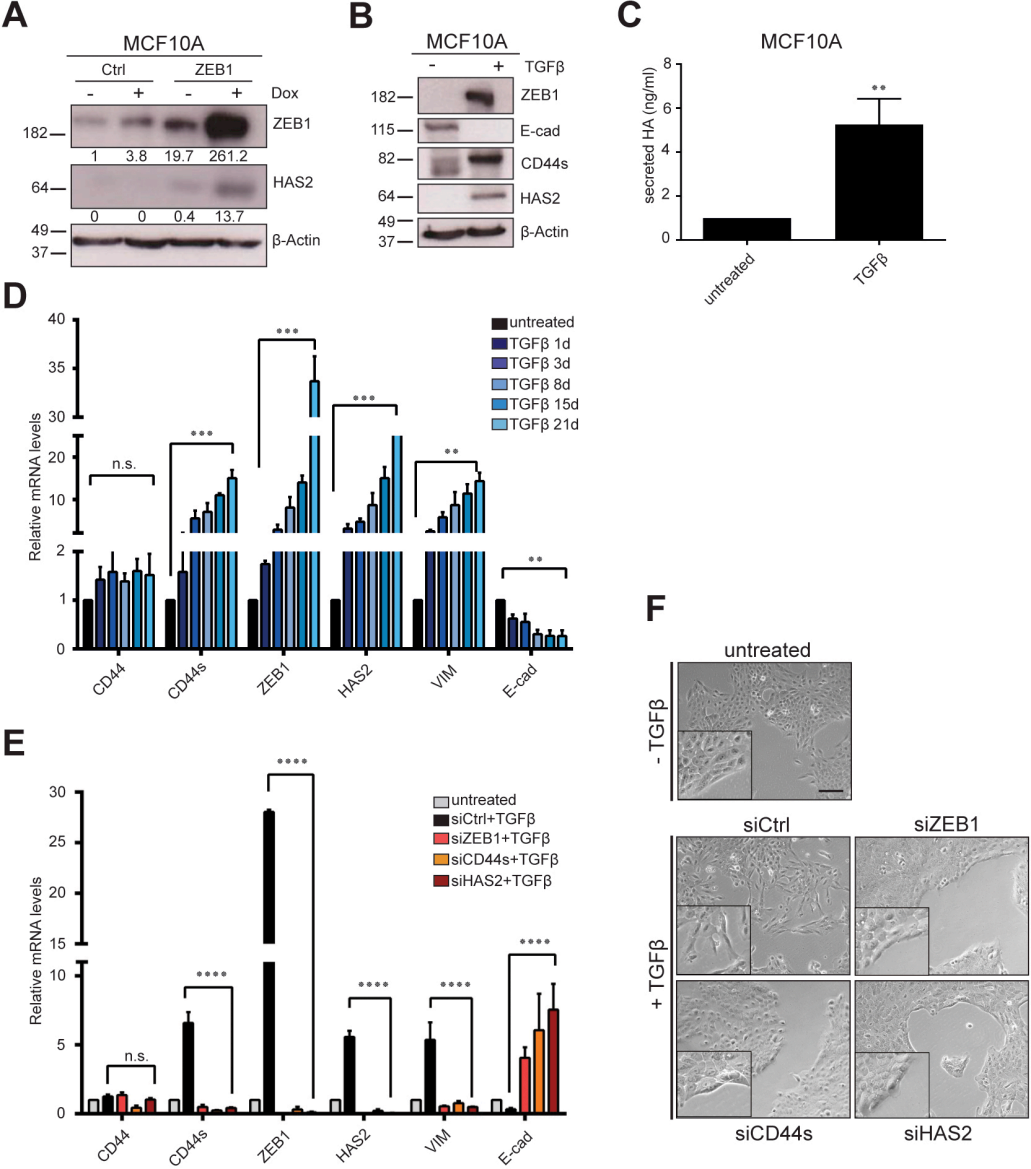
HAS2 becomes activated during EMT along with common EMT markers (**A**) MCF10A cells stably transfected with a tetracycline-responsive construct for inducible *ZEB1* expression, treated with 1 μg/ml doxycycline (Dox) for six days. Western blot shows induced ZEB1 and upregulation of HAS2 upon Dox treatment. Quantification of blots are given below the individual blots, showing a > 10-fold upregulation of ZEB1 in ‘ZEB1’ cells upon Dox treatment as well as a weak Dox-dependent activation of endogenous ZEB1 in ‘Ctrl’ cells. (**B**) EMT induction of wildtype MCF10A cells by TGFβ treatment for 21 days analyzed by Western blot. E-cad is downregulated while CD44s and HAS2 are activated. (**C**) Treatment of MCF10A cells with TGFβ increases secretion of HA. (**D**) Time-course experiment of TGFβ treatment of MCF10A cells shows a gradual increase of *HAS2* levels that follow the increase in *ZEB1*, *vimentin* and *CD44s* transcripts by qRT-PCR analysis. Simultaneously, *E-cad* is downregulated and *CD44 total* levels remain unchanged. (**E**) Knockdown of *HAS2* prevents EMT in TGFβ-treated MCF10A cells. qRT-PCR of transcript levels upon 21-days TGFβ treatment and simultaneous knockdown of *ZEB1*, *CD44s* or *HAS2*. Similar to siZEB1 and siCD44s transfection, siHAS2 prevents activation of *vimentin*, *CD44s* and *ZEB1* and *E-cad* is even increased in comparison to siCtrl samples. (**F**) Phenotypically, cells are prevented from EMT and stay clustered in all transfectants, except for siCtrl samples. Scale bar, 200 μm.

To gain further insights into the dynamic changes of HAS2 and ZEB1 levels during EMT and how they control each other's expression after initiation of EMT, we utilized *in vitro* systems to induce EMT. MCF10A cells are fibrocystic mammary epithelial cells without tumorigenic potential *in vivo*, but they can undergo EMT upon TGFβ stimulation or by induction of exogenous *ZEB1* expression [19, 40]. Experimental induction of *ZEB1* from a stably transfected doxycycline (Dox)-inducible expression construct in MCF10A cells raised the levels of HAS2 protein after seven days of Dox treatment, but not in empty vector control cells (Figure 3A). Long-term TGFβ stimulation of wildtype MCF10A cells for 21 days had similar effects. During EMT induction, ZEB1 levels were increased, whereas E-cad became downregulated. Concomitantly, HAS2 expression and CD44s splicing were induced, as evident on protein level (Figure 3B). In line with increased HAS2 levels by TGFβ treatment, secreted HA was elevated (Figure 3C). We next analyzed MCF10A cells in shorter intervals during the 21 days of TGFβ treatment to observe dynamic changes in gene expression during EMT by qRT-PCR. In agreement with the endpoint analysis and our previous observations, we found a gradual increase of *CD44s*, *ZEB1*, *vimentin* and a reduction of *E-cad* transcripts, whereas total *CD44* levels remained constant, presumably due to alterations in ESRP1-regulated splicing (Figure 3D) [19, 41]. *HAS2* showed a similar gradual increase of transcript levels over the entire duration of TGFβ treatment and the fold-changes between *ZEB1* and *HAS2* were of the same range, suggesting that their expression is linked and regulated by a common mechanism (Figure 3D). Knockdown of *HAS2* in MCF10A cells during the 21 days of TGFβ treatment resulted in a complete block of EMT. *ZEB1* levels were not increased, *CD44* differential splicing was not shifted towards *CD44s* and *E-cad* was not downregulated. In comparison to untreated cells, siRNA mediated *HAS2* knockdown inverted the effects of TGFβ and even pushed the cells towards a more epithelial phenotype with reduction in *ZEB1*, *vimentin* and *CD44s* and increased *E-cad* levels at the end of the treatment (Figure 3E). Knockdown of *HAS2* during TGFβ treatment changed also the shape of the cells to a cobblestone-like morphology and formation of epithelial clusters, whereas siCtrl transfection did not affect formation of a spindle-shape morphology and single cell spreading (Figure 3F). Similar effects were observed upon siZEB1 and siCD44s transfections. These results indicate that both ZEB1 and HAS2 are essential for TGFβ-induced EMT and their expression is co-regulated, presumably interdependent.

### ZEB1 is activating *HAS2* expression by binding to the *HAS2* promoter

**Figure 4:**
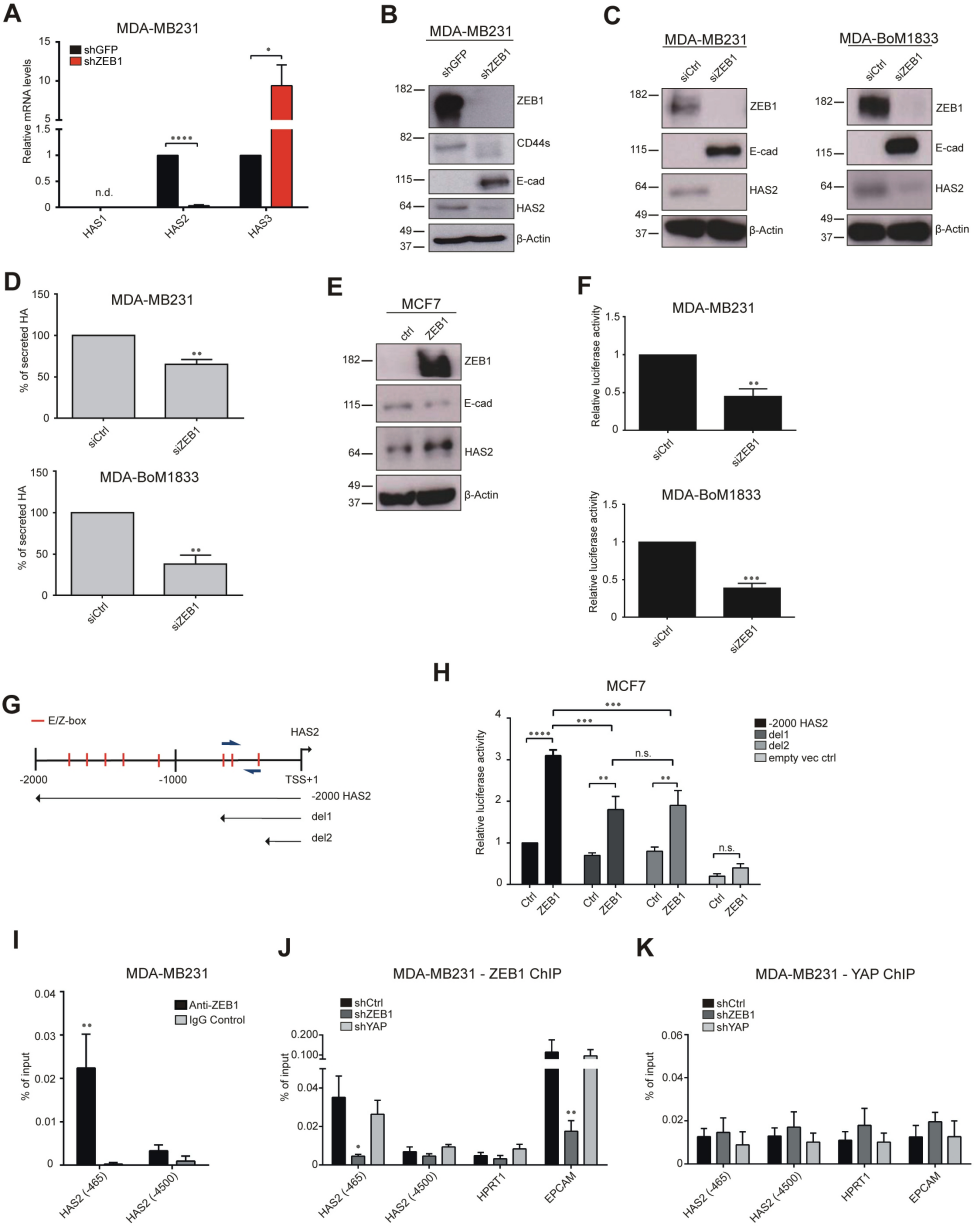
ZEB1 directly regulates HAS2 expression (**A**) mRNA analysis of *HAS1*, *HAS2* and *HAS3* levels in MDA-MB231 cells after stable *ZEB1* knockdown shows a specific loss of *HAS2* transcripts whereas *HAS3* is even upregulated. *HAS1* is not detected (n.d.) in shGFP and shZEB1 samples. (**B**) Western blot analysis of a stable knockdown of *ZEB1* in MDA-MB231 cells. (**C**) Western blot of cells with a transient knockdown of *ZEB1* in MDA-MB231 and MDA-BoM1833 cells. Knockdown of *ZEB1* substantially reduces HAS2 expression. (**D**) Transient *ZEB1* knockdown reduces levels of secreted HA in MDA-MB231 and MDA-BoM1833 cells. (**E**) Overexpression of *ZEB1* in MCF7 cells induces HAS2 upregulation and E-cad reduction observed by Western blot. (**F**) Luciferase assay in MDA-MB231 and MDA-BoM1833 cells transiently transfected with a -2000 bp to +1 bp promoter fragment of the human *HAS2* locus cloned 5-prime of a luciferase reporter gene. Knockdown of *ZEB1* reduces the reporter gene activity. (**G**) Schematic representation of the human *HAS2* promoter region between -2000 and +1 bp relative to the transcription start site (TSS). Red lines indicate positions of canonical E- and Z-boxes and primer locations used for ChIP are indicated by blue arrows. Sequences included in deletion constructs containing three proximal E-boxes (del1) and no E-boxes (del2) are indicated by arrows. (**H**) Transient transfection of MCF7 cells with the full-length *HAS2* promoter construct, del1 or del2 reveal that co-transfected *ZEB1* is activating the full-length construct and to lesser extend the two deletion constructs. (**I**) Chromatin immunoprecipitation (ChIP) using anti-ZEB1 and control IgG in MDA-MB231 cells. Enrichment of ZEB1 is seen at the proximal promoter (−465), but not at a distal region of the locus (−4500). (**J**, **K**) ChIP of the *HAS2* promoter in MDA-MB231 shCtrl, shZEB1 and shYAP stable knockdown cells with anti-ZEB1 (J) and anti-YAP (K) antibodies, showing that ZEB1-specific DNA precipitation is lost at *HAS2* (−465), similar to the promoter of the known ZEB1 target gene *EPCAM*, whereas ZEB1 binding is unaffected by YAP knockdown.

ZEB1 regulates various genes, like miR-200 family members, *ESRP1* and others in feedback loops [12, 14, 19]. To assess whether *HAS2* expression is also directly controlled by ZEB1, we analyzed *HAS2* levels upon *ZEB1* knockdown and ZEB1 occupancy at the *HAS2* locus. In stable shZEB1 knockdown clones of MDA-MB231 cells [42] *HAS2* expression was decreased > 100-fold, whereas that of *HAS3* was 5-fold increased. *HAS1* was not detected in shGFP or shZEB1 knockdown cells (Figure 4A). The decrease in *HAS2* levels was confirmed by Western blotting (Figure 4B). To distinguish between short and long-term effects, we transiently knocked down *ZEB1* in wildtype MDA-MB231 and MDA-BoM1833 cells and observed a similar reduction of HAS2, combined with E-cad upregulation upon *ZEB1* knockdown (Figure 4C). Simultaneous to the loss of HAS2, secreted HA was reduced upon siZEB1 transfection in MDA-MB231 and MDA-BoM1833 cells (Figure 4D). *Vice versa*, overexpression of *ZEB1* in the epithelial MCF7 breast cancer cell line resulted in downregulation of E-cad and a slight activation of HAS2 (Figure 4E). To understand whether ZEB1 directly binds to the *HAS2* promoter, we cloned a -2000 to +1 bp fragment of the human *HAS2* locus upstream of a luciferase reporter gene. Knockdown of *ZEB1* in MDA-MB231 and MDA-BoM1833 cells resulted in reduced activity of the *HAS2*-luciferase reporter construct (Figure 4F). A closer inspection of the promoter region identified eight E-boxes as putative binding sites for ZEB1 in this construct. Five of them were located between -2000 and -1000 bp and three were identified at positions -329, -522 and -563 bp (Figure 4G). We generated deletion constructs del1 and del2 that harbor the three proximal E-boxes and no E-boxes, respectively (Figure 4G). In MCF7 cells the full-length construct revealed a 3-fold activation upon transient *ZEB1* overexpression (Figure 4H). This effect was reduced if regions harboring five or all eight E-boxes were deleted. However, a moderate 2-fold upregulation of the luciferase reporter was observed with the del2 construct that was not completely diminished in the empty vector control (Figure 4H). As we have shown previously that ZEB1 is interacting with YAP to activate specific common target genes [9], HAS2 might be activated by a similar E-box independent mechanism. Chromatin immunoprecipitation of the endogenous *HAS2* locus in MDA-MB231 cells identified a substantial enrichment of ZEB1 at the promoter region of *HAS2* (−465 bp) in contrast to a random distal region at -4500 bp (Figure 4I). This enrichment was lost in *ZEB1* knockdown cells, similar to the known ZEB1 target gene *EPCAM* (Figure 4J). However, we could not identify a specific YAP binding to the *HAS2* locus, as *YAP* knockdown did not result in decreased ZEB1-mediated precipitation of the *HAS2* promoter (Figure 4J) and anti-YAP ChIP did not show enrichment in shCtrl vs. shYAP cells (Figure 4K). Interestingly, HAS2 levels were reduced 2-fold upon *YAP* knockdown, although not significantly (Supplementary Figure S5). These results indicate that *HAS2* expression which is the main driver of HA production in the analyzed breast cancer cell lines is directly controlled by ZEB1, thereby linking initial EMT signals to the secretion of excess HA.

### Extracellular HA and conditioned medium of MDA-MB231 and MDA-BoM1833 cells accelerates osteoclast differentiation in a HAS2-dependent manner

**Figure 5:**
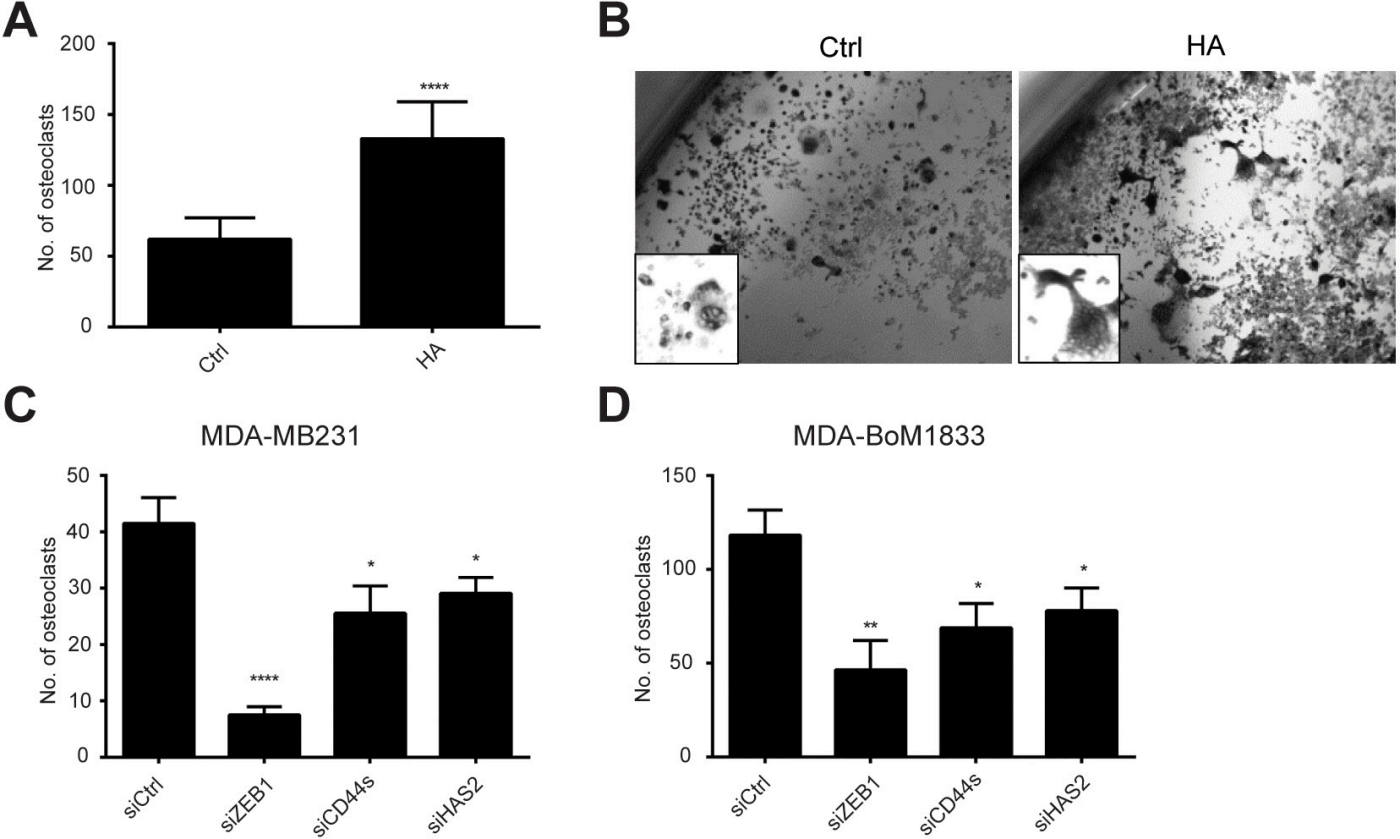
HA-enriched conditioned medium of MDA-MB231 and MDA-BoM1833 cells induces osteoclast differentiation of Raw264.7 cells to osteoclasts (**A**, **B**) Quantification (A) and morphology (B) of tartrate-resistant acid phosphatase stained osteoclasts in cultures of Raw264.7 cells under differentiation conditions with RANKL (Ctrl). Differentiation is enhanced 2.5 fold by additional HA treatment (HA). (**C**, **D**) Conditioned medium of MDA-MB231 (C) and MDA-BoM1833 cells (D) is providing differentiation cues for osteoclast formation of Raw264.7 cells. Knockdown of *ZEB1*, *CD44s* and *HAS2* reduces the capacity of the conditioned medium to induce osteoclast differentiation.

The life-threatening event during tumor progression downstream of EMT is the formation of metastases. It was previously shown that HAS2 is crucial for creating a pro-metastastic microenvironment allowing triple-negative breast cancer cells to efficiently colonize to the bone [34, 43]. This is in part attributed to enhanced osteolysis by increased osteoclast differentiation which is stimulated by HAS2 and HA [44]. We wondered whether the ZEB1-dependent induction of *HAS2* and thus increased HA secretion enhances osteoclasts differentiation in support of formation of a pro-metastatic niche. Therefore we used murine monocyte-macrophagic Raw264.7 cells, a well-established *in vitro* model of osteoclast differentiation. In presence of RANKL Raw264.7 cells start to form osteoclasts within one week, identified by tartrate-resistant acid phosphatase (TRAP) staining (Figure 5A and 5B). When these cells were incubated with HA in addition to RANKL, the amount of differentiated osteoclasts was more than doubled from 60 to 130 in a 6-well plate, indicating that external supply of HA is promoting osteoclast differentiation (Figure 5A and 5B). Importantly, when Raw264.7 cells were incubated with supernatant of MDA-MB231 or MDA-BoM1833 cells in which *ZEB1* was knocked down, the amount of osteoclasts was significantly reduced to 10% in comparison to incubation with control supernatant. A similar but less pronounced effect was observed when *CD44s* or *HAS2* expression was silenced by siRNA in MDA-MB231 or MDA-BoM1833 cells (Figure 5C and 5D). These results demonstrate that tumor cell derived HA promotes osteoclast differentiation and that the effect is dependent on HAS2, CD44s and ZEB1 activities.

## DISCUSSION

Besides the deregulation of key signaling pathways, tumor progression is dependent on the ability of tumor cells to recruit and instruct the microenvironment for providing pro-tumorigenic cues. These cues are required to promote tumor growth, survival, evasion from immune surveillance and metastatic spread by EMT induction [21]. The interaction with the microenvironment involves remodeling of the extracellular matrix, including increased deposition of different proteoglycans such as hyaluronic acid into the extracellular space [20]. We found tumor cell-derived HA to promote EMT, presumably by binding to CD44s. While excess HA induced *ZEB1* expression, blocking HA synthesis reduced *ZEB1* levels in triple-negative breast cancer cells. Expression of *ZEB1* and *HAS2*, the main synthase of HA in tumor cells, was strongly correlated in breast cancer cell lines and tumor patient samples and high expression levels of *HAS2* were associated with poor survival. Interestingly, ZEB1 was found to bind to the *HAS2* promoter and to activate its expression. This suggests another level of maintaining an EMT state of tumor cells by enforcing the autocrine HA production via ZEB1-mediated transcriptional activation of *HAS2*. HA secretion by tumor cells affected not only tumor cells but also the pro-metastatic niche. We found that conditioned medium from MDA-MB231 and even stronger from the selected bone-metastatic subline MDA-BoM1833 enhanced the differentiation of monocytes to osteoclasts. This is of particular interest for the formation of macrometastases as tumor cells have to remodel the bone for efficient colonization [45, 46]. Accordingly, tumor cells-derived HA likely interferes with bone homeostasis by shifting the equilibrium of bone generating osteoblasts and osteolytic osteoclasts towards increased osteoclast numbers supporting bone destruction. In line with our findings, HAS2 was shown to promote tumor growth and metastases in bones by stimulating the interaction of breast cancer stem-like cells with macrophages and stromal cells [43]. Similarly, inactivating CD44 as the main HA receptor by shRNA knockdown blocked metastatic spreading of MDA-MB231 cells injected into the mammary fat pad of immunodeficient mice. The reduction of bone metastases correlated with a decrease in the number of osteolytic osteoclasts [44].

In the differentiation experiment of Raw264.7 cells conditioned medium of MDA-MB231 or MDA-BoM1833 cells with a *ZEB1* knockdown reduced osteoclast differentiation more efficiently than knockdown of *HAS2* and *CD44*. ZEB1 was shown to induce the expression of the BMP inhibitors NOG, FOL and CHRDL1, which are secreted and known to promote osteolysis [11, 47]. Hence, HA and BMP inhibitors cooperate in bone remodeling. Accordingly, knockdown of *ZEB1* in the *in vitro* differentiation system had a stronger effect, because it is blocking *HAS2* expression and HA secretion and is reducing BMP-inhibitor accumulation in the conditioned medium simultaneously.

HA synthesis by HAS1-3 and its deposition into the ECM is increased during EMT and is known to play a crucial role during tumor progression [20]. Recently, the corresponding synthase genes in mice, in particular *Has2* and *Has3*, were found among the most highly upregulated genes during the early phase of TGFβ-induced EMT. This was dependent on Smad4 and the tumor suppressive function of TGFβ in a mouse model of pancreatic cancer [48]. These findings support our idea that *HAS2* is induced during EMT by ZEB1 and that its production of HA is crucial for sustaining EMT signals.

Of note, it was demonstrated that HAS2 supports tumorigenesis mainly by its synthase activity of HA [20]. However, an HA-independent function of HAS2 supporting tumor progression was suggested by recent findings. Blocking newly synthesized HA or blocking HA-CD44 interaction did not efficiently block EMT in TGFβ-treated NMuMG, whereas knockdown of *Has2* completely abolished EMT [49]. Whether an intracellular HA-synthase-independent function of HAS2 is active in our cellular models could not be sufficiently addressed. However, we observed strong effects on ZEB1 levels and EMT when HA synthesis was blocked by 4-MU. In line with this, CD44s seems to be required for the effect of HA on *ZEB1* expression, but how HA and CD44s then mechanistically act on *ZEB1* transcription remains elusive. Very likely, HA binds to CD44s which then induces signaling by the receptor [26].

Although our results and several other studies indicate a pro-tumorigenic function of HA, there are also conflicting data that support a more anti-tumorigenic function. In squamous cell carcinoma decreased HA levels were associated with poor survival. Similarly, in cutaneous melanoma reduced HA and CD44 levels lead to an early tumor relapse and poor survival [50, 51]. These effects might be related to different functions of low, medium and high molecular weight HA in a context-dependent manner. As an example: high molecular weight HA acts mainly anti-angiogenic as it inhibits endothelial proliferation and migration *in vitro* [20]. However, *in vivo* experiments showed that it supports angiogenesis presumably by interacting with different ECM components like FGFs and proteoglycans [52]. This shows that the function of HA is very complex. Moreover, the balance between HA synthesis and degradation controls the cellular responses. It will be interesting to further explore on this equilibrium in breast cancer to obtain a comprehensive view on how HA triggers EMT, invasion and metastasis.

Recent findings support the notion that ZEB1 not only acts as a transcriptional repressor to suppress epithelial-specific genes like *E-cad* and members of the miR-200 family, but can also activate transcription in specific contexts by interacting with different co-factors [9, 11]. Interestingly, this function of ZEB1 does not necessarily require direct binding of ZEB1 to the DNA. The activation of a specific ZEB1/YAP target gene set seems to be independent of any canonical ZEB1 DNA-binding motif, like Z- or E-boxes at the target gene promoters [9]. In our analysis, a reduced expression of a *HAS2* luciferase reporter construct was detected when proximal E-box motifs were deleted. However, ZEB1 was still able to partially activate the construct in absence of a conserved E-box. Although we have not formally proven that ZEB1 is not directly interacting with the DNA of the *HAS2* promoter, a similar mechanism as for common ZEB1/YAP target genes may be active to drive *HAS2* expression. It will be interesting to further dissect how ZEB1 is activating the promoter and which transcription factors are involved.

In summary, our results provide novel insights into how ZEB1 utilizes HAS2/HA to enforce its own expression and to shape the microenvironment. This crosstalk is used as amplifying module to support the previously identified ZEB1/ESRP1/CD44s feedback loop [19]. Initial external EMT-stimuli activate *ZEB1* expression that simultaneously provides ligand (HA) and generation of the corresponding receptor (CD44s) to further accelerate EMT. Activation of (1) *CD44* differential splicing by ESRP1-loss and (2) activation of HA synthesis by direct regulation of *HAS2* help to maintain high *ZEB1* expression. In conclusion, together with our previous findings, the analysis provides insights into a complex multi-factorial feedback system controlled by ZEB1 to induce EMT and metastatic behavior of breast cancer cells.

## MATERIALS AND METHODS

### Cell culture

MDA-MB231, MCF7, MCF10A and monocyte-macrophagic Raw264.7 cell lines were purchased from ATCC and MDA-BoM1833 were kindly provided by Joan Massagué (Sloan-Kettering Institute for Cancer Research, New York). MDA-MB231 shGFP and shZEB1 stable knockdown clones have been described previously [42] and MDA-MB231 shCtrl and shYAP knockdown cells were generated by lentiviral transduction of pGIPZ constructs harboring a non-silencing control (RHS4346) and V3LHS-306099, respectively. Non-transduced cells were eliminated by puromycin selection (2.5 μg/ml) for 3 days. Cells were cultured in DMEM/10% FCS or in DMEM/F12 (Invitrogen, 10566 and 31331) supplemented with 5% horse serum (Life Technologies, 16050122), 20 ng/ml EGF (R&D Systems, 236EG200), 0.5 μg/ml hydrocortisone (Sigma, H0888), 0.1 μg/ml cholera toxin (Sigma, C-8052) and 10 μg/ml insulin (Invitrogen, 12585–014) for MCF10A cells. EMT was induced by treatment with 5 ng/ml TGFβ1 (PeproTech, 100-21) for the indicated time replacing the medium every other day. Induction of *ZEB1* expression in MCF10A cells was induced by adding 1 μg/ml doxycycline (Dox, Sigma, D9891) every other day as described previously [19, 53]. Inhibition of HAS1-3 to block HA synthesis was performed by treatment of cells with indicated concentrations of 4-Methylumbelliferone (4-MU, Sigma, M1381) for 72 h. Hyaluronic acid sodium salt from Streptococcus equi bacterial glycosaminoglycan polysaccharide (HA, Sigma, 53747-1G) or Sodium Hyaluronate 5000 (Healon, 10-2000-12) was dissolved in H_2_O and used at a final concentration of 250–400 μg/ml in DMEM/10% FCS for 24 h. All cells were kept at 37°C, 5% CO_2_ in a humidified incubator.

### Western blotting

Cells were rinsed once in PBS and lysed in TLB. 30 μg of protein was separated by SDS-PAGE (10%) for 1 h, 150 V and transferred to a nitrocellulose membrane by wet blotting in transfer buffer for 2 h, 300 mA at 4°C. Membranes were immersed in Antigen pretreatment solution (SuperSignal Westernblot Enhancer, Thermo Scientific) for 10 min and blocked in 5% skim milk/TBST) for 30 min at room temperature. Primary antibody incubation was carried out in Primary antibody Diluent (SuperSignal Westernblot Enhancer, Thermo, 46641) over night at 4°C. After washing in TBST, membrane was incubated with HRP-conjugated secondary antibody in 5% skim milk/TBST for 1 h at RT. Detection was carried out using SuperSignal West Femto Maximum Sensitivity Substrate (Thermo, 34094) or ECL Prime Westenblot Detection Reagent (Amersham, RPN2232) and a ChemiDoc imaging system (BioRad). Quantification was performed where appropriate using ImageJ and presented normalized to β-Actin levels.

### Antibodies

The following antibodies and dilutions were used for Western blotting: mouse anti-E-cadherin (BD Pharmingen, 610181; 1:5,000), mouse anti-β-Actin (Sigma, A5441; 1:5,000), mouse anti-CD44 (R&D Systems, BBA10; 1:1000), mouse anti-HAS2 (Abcam, H00054845-B01P; 1:500) and rabbit anti-ZEB1 (Sigma, HPA027524; 1:5,000), as well as HRP-coupled goat anti-rabbit IgG (Dianova, 111-035-003; 1:25,0000) and goat anti-mouse IgG (Dianova, 115-035-003; 1:25,000).

### Total RNA isolation and quantitative reverse transcriptase PCR (qRT-PCR)

Total RNA was isolated using the RNeasy Plus Mini Kit (Qiagen, 74136) according to manufacturer's protocol. cDNA was synthesized from 1 μg total RNA with the RevertAid First Strand cDNA Synthesis Kit (Thermo, K1622), using 0.5 μl oligo (dT) and 0.5 μl random hexamer primers. Transcript levels were analyzed by qRT-PCR using cDNA amounts corresponding to 7.5 ng of original total RNA and 300 nM primers (see Supplementary Table S1) with the Power SYBR Green PCR Master Mix (Applied Biosystems, 4368702) in a LightCycler 480 (Roche). Relative expression levels were calculated and normalized to those of *ACTB* applying the Pfaffl method.

### Indirect immunofluorescence labeling

Cells were plated on cover slips, fixed with 1% PFA/PBS, followed by permeabilization step with 0.25% Triton X-100/PBS (10 min each). After blocking in 3% BSA/PBS for 30 min, antibodies were diluted 1:200 in blocking solution and applied for incubation overnight. Alexa488 and −594 conjugated antibodies were used in same dilutions for 45–60 min. Cover slips were mounted with Citifluor/1 μg/ml DAPI and imaged on a Leica DM5500 microscope.

### Generation of reporter gene constructs and luciferase assay

2 kb of the *HAS2* promoter region containing E-Boxes at −329, −522, −563, −1065, −1397, −1508, −1613 and −1743 bp as well as del1 (−702 to +1 bp) and del2 (−248 to +1) sequences were amplified by *Pfu*Ultra HF polymerase (Agilent, 600380-51) with forward1 (5′-ataatgagctcaacaacaaatgtgtttttct-3′), forward2 (5′-ataatgagctcccacggcagaaacctcttta-3′) or forward3 (5′-ataatgagctccggcctgtagctcagagaag-3′) and reverse (5′-ataatagatcttccttccccgccgttgttgc-3′) primers and cloned 5′ of the luciferase gene into pGL4.10 (Promega) by *Xho*I/*Xba*I using standard molecular cloning techniques. For the luciferase reporter assay, cells were seeded in 24-well plates and transfected 24 h later with siRNAs (see Supplementary Table S2) and with plasmid DNA another 48 h later. The second transfection contained 50 ng pRL-TK, 300 ng luciferase reporter construct and 150 ng pCIneo-ZEB1 or pCIneo empty vector control. Cells were harvested 24 h after plasmid transfection and lysed in 100 μl passive lysis buffer for 15 min and measured by using the Dual-Luciferase^®^ Reporter Assay System (Promega, E1910) according to the manufacturer's instructions. Firefly luciferase levels were normalized to Renilla luciferase levels and the activity of the −2000 *HAS2* promoter construct alone was set to one. Experiments were repeated three times.

### Transfection of plasmid DNA and siRNA

Plasmid DNA transfection was done by using the FugeneHD transfection reagent (Promega, E2311) according to the manufacturer's instructions. siRNAs were purchased from Ambion and sequences are provided in Supplementary Table S2. 0.4 mM of individual siRNAs were transfected with Lipofectamine RNAiMax (Invitrogen, 13778075) and unless otherwise indicated harvested 72 h afterwards for protein or RNA analysis as previously described [19].

### Chromatin immunoprecipitation

ChIP was performed as previously described [11]. 500 μg of chromatin was incubated with 5 μg of anti-ZEB1 (Santa Cruz, H102, sc-25388X) and rabbit IgG control (Santa Cruz, sc-2345) antibodies at 4°C overnight. 25 μl of a 1:1 ratio of protein A/G Dynabeads (Invitrogen,10002D, 10004D) were used to precipitate antibody-bound chromatin. Upon elution with 0.1 M NaHCO3, 1% SDS and decrosslinking with 250 μg/ml RNaseA and 500 μg/ml proteinase K at 65°C overnight, specific genomic regions were analyzed by qPCR. Primers are given in Supplementary Table S3.

### Statistical analysis

Statistical analyses were performed using GraphPad Prism 6 (GraphPad Software, Inc.). Normalized relative expression levels were used to calculate the mean and the SEM for *n* = 3 unless otherwise stated. Statistical significance was evaluated by two-tailed Student's *t*-test and multi-group comparisons by 2-way ANOVA and Tukey's post-hoc test was used when appropriate. *p*-values of statistical significance are given: *p < 0.05; **p < 0.01;***p < 0.001; ****p < 0.0001.

### Correlation analysis and Kaplan-Meier plot

Expression data for the ‘CCLE panel’ of cancer cell lines comprising 36 cancer types from 917 cancer cell lines, the ‘NCI60 panel’ and of 275 lung, 104 breast and 45 pancreatic cancer patients, including follow-up data, were downloaded from the NCBI GEO database (GSE5846, GSE36133, GSE41271, GSE42568, GSE28735, GSE2034, GSE18229) [35–39, 54, 55]. Correlation analysis was done using GraphPad Prism. Relapse and overall survival was compared between patients with high and low *HAS2* expression levels with a cut-off at the average expression values above the 58th percentile and below the 42th percentile. Kaplan-Meier plotting and log-rank test was used to evaluate the differences in survival between defined groups.

### Immunohistochemistry

Samples were retrieved from local archives and usage was approved by the Ethics Committee of the University of Freiburg. Immunohistochemistry was performed as described previously [9] using paraffin embedded tissue, sectioned to 4 μm, anti-ZEB1 (Sigma, #HPA027524; 1:800), anti-HAS2 (Abcam, H00054845-B01P; 1:200) antibodies and the EnVision-System (DAKO, K4003, K4001). Antigen retrieval was carried out on deparaffinized and rehydrated sections in 10 mM citrate buffer, pH 6.0 in a pressure cooker and DAB substrate (Thermo, 002020) was used for visualization of antibody binding. Slides were counterstained with Mayer's hematoxylin (Merck, 1.09249.0500) and mounted with Histokitt (Roth, 6640).

### Osteoclast differentiation assay

The secretome of individual cell lines and transfectants was analyzed to promote osteoclast differentiation of Raw264.7 cells as previously described [11]. In brief, supernatant of MDA-MB231 and MDA-BoM1833 transfected with siRNAs was collected after 24 h. One thousand Raw264.7 cells per well were seeded in a 96-well plate and incubated with cell supernatants supplemented with 5 ng/ml RANKL (PreproTech, 315-11) as indicated with or without 250 ng/ml hyaluronic acid. Tartrate-resistant acid phosphatase (TRAP) staining was done with the leukocyte acid phosphatase kit (Sigma, 387A-1KT) one week afterwards to access the number of osteoclasts.

### Enzyme-linked immunosorbent assay (ELISA)

The amount of secreted HA was analyzed by Quantikine ELISA Hyaluronan KIT (R&D, DHYAL0). Cells were serum-starved for 24 h and medium was collected and processed according to the manufacturer's instructions.

## SUPPLEMENTARY MATERIALS FIGURES AND TABLES


