# Supplementary material for: A novel ZEB1/HAS2 positive feedback loop promotes EMT in breast cancer
Source: Oncotarget. 2017 Jan 9;8(7):11530–43. doi: 10.18632/oncotarget.14563 (PMC5355283; doi:10.18632/oncotarget.14563)
Supplement: Supplementary file 1 [file oncotarget-08-11530-s001.pdf]

A novel ZEB1/HAS2 positive feedback loop promotes EMT in breast cancer

Supplementary Materials

Supplementary Table S1: Primers used in qRT-PCR analysis

Gene	forward primer (5′ - 3′)	reverse primer (5′ - 3′)
ACTB	GCCCTGAGGCACTCTTCCA	TTGCGGATGTCCACGTCA
CDH1	GTCCTGGGCAGAGTGAATTT	GACCAAGAAATGGATCTGTGG
VIM	CGAGGAGAGCAGGATTTCTC	GGTATCAACCAGAGGGAGTGA
ZEB1	AAGAATTCACAGTGGAGAGAAGCCA	CGTTTCTTGCAGTTTGGGCATT
CD44 total	ATAATTGCCGCTTTGCAGGTGTATT	ATAATGGCAAGGTGCTATTGAAAGCCT
CD44s	ATAATAAAGGAGCAGCACTTCAGGA	ATAATTGTGTCTTGGTCTCTGGTAGC
HAS1	TGCTCAGCATGGGTTATGC	AGGGCGTCTCTGAGTAGCAG
HAS2	CTCCGGGACCACACAGAC	TCAGGATACATAGAAACCTCTCACA
HAS3	ACCATCGAGATGCTTCGAGT	CCATGAGTCGTACTTGTTGAGG

Supplementary Table S2: siRNA sequences

Name (Gene)	sequence (5′ - 3′)
**siZEB1**	GGUAGAUGGUAAUGUAAUAtt
**siCD44s**	CCUGCUACCAGAGACCAAGtt
**siHAS1**	GGAAUAACCUCUUGCAGCAtt
**siHAS2**	GUAUCUGCAUCAUGCAAAAtt
**siHAS3**	ACCUGUCCUUCGGCCUGUAtt
**siCtrl**	GCUACCUGUUCCAUGGCCAdTT

Supplementary Table S3: Primers used in ChIP experiments

Position rel. to HAS2 TSS	forward primer (5′ - 3′)	reverse primer (5′ - 3′)
–465	ACACACACACGTGCACACAC	GCGTTACAAGTTCCCTCCAA
–4500	GGAGATCGAGACCATCTTGG	CCCGAGTAGCTGGGACTACA

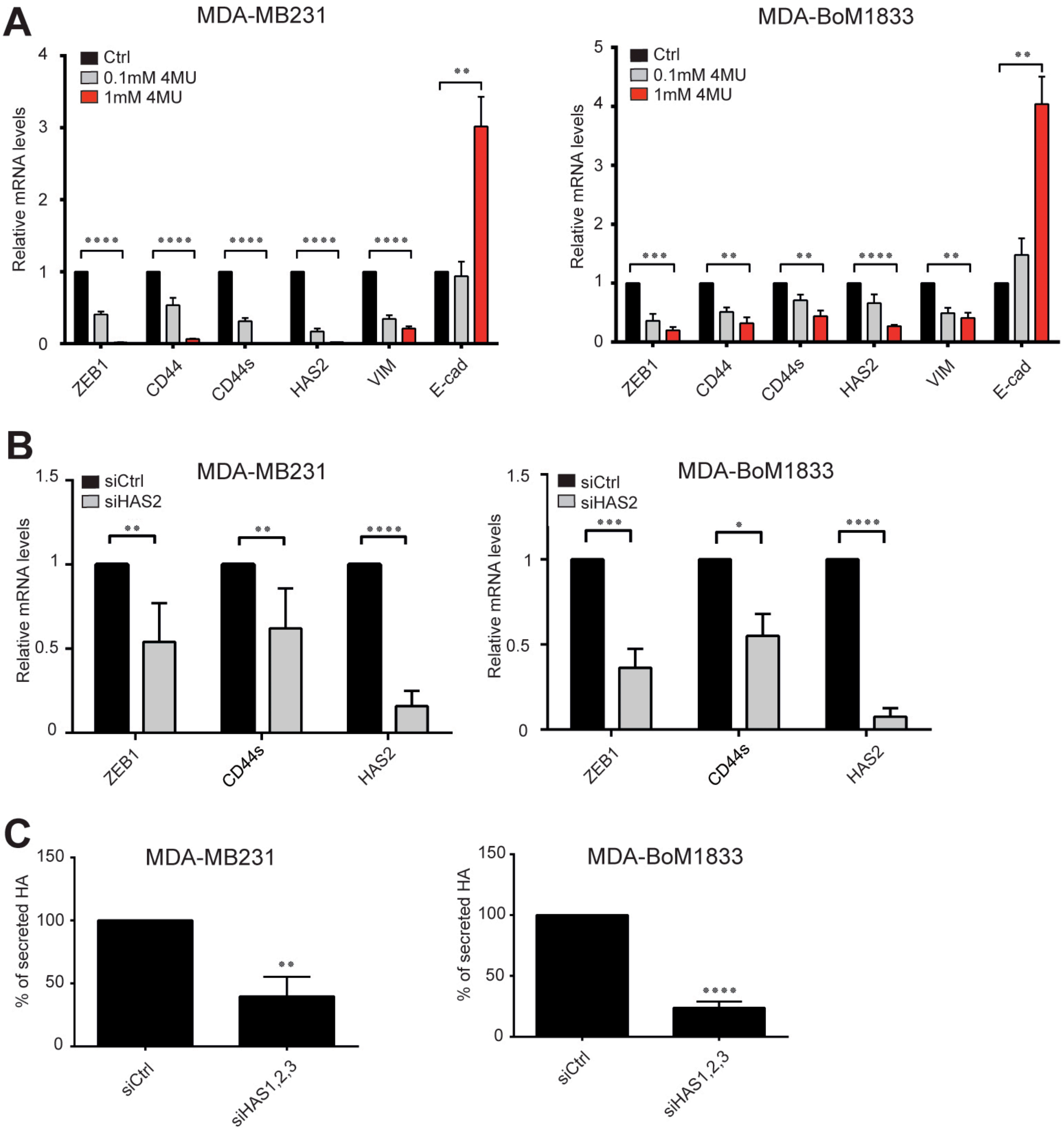

Supplementary Figure S1: Blocking of HA generation and secretion is preventing EMT in triple-negative breast cancer cells MDA-MB231 and MDA-BoM1833. (**A**) qRT-PCR upon treatment with 0.1 mM and 1 mM 4-MU leads to a reduction in *ZEB1*, *vimentin* and *CD44s* transcripts, whereas *E-cad* is increased in a concentration-dependent manner indicating MET. (**B**) Knockdown of *HAS2* is reducing *ZEB1* and *CD44s* transcript levels in qRT-PCR quantifications, similar to 4-MU treatment. (**C**) Analysis of secreted HA levels upon combined knockdown of *HAS1*, *HAS2* and *HAS3* reveals only moderate additional reduction of HA secretion in comparison to single *HAS2* knockdown (Figure 1G).

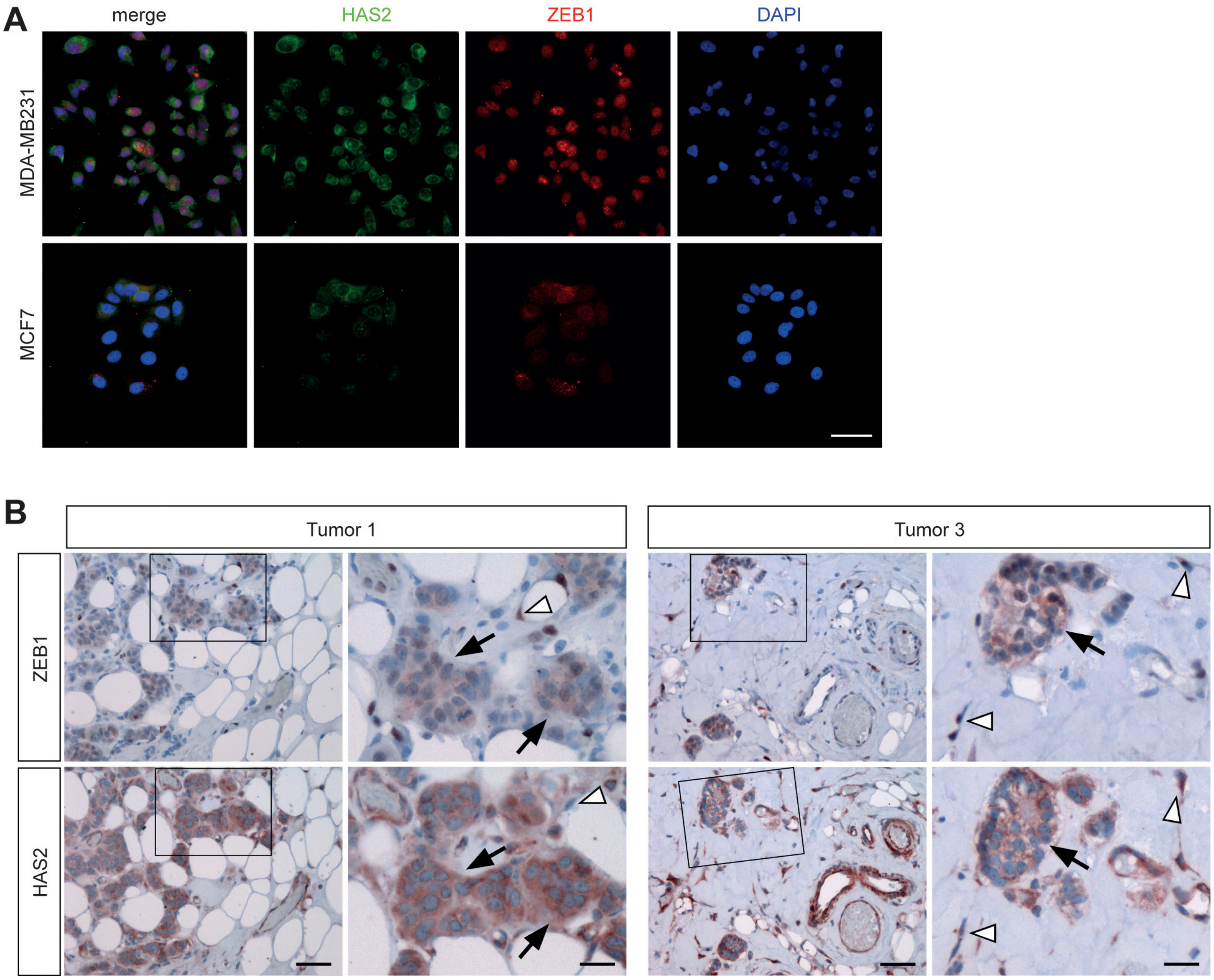

Supplementary Figure S2: HAS2 and ZEB1 expression is correlated in breast cancer cell lines MDA-MB231 and MCF7 and in ZEB1-positive breast tumors. (**A**) Indirect immunofluorescence labeling of HAS2 (green) and ZEB1 (red) in MDA-MB231 (upper panel) and MCF7 cells (lower panel). MDA-MB231 cells show ZEB1 staining in the nucleus and cytoplasmic HAS2 localization, whereas MCF7 cells are negative for both proteins, displaying only a relatively strong unspecific background staining of the HAS2 antibody. Scale bar, 50 µm. (**B**) Sections of additional tumor samples, complementing the analysis of Figure 2D. Both samples show expression of ZEB1 in tumor cells, although not exclusively nuclear (Tumor 1). Areas of tumor cell clusters that are ZEB1 positive (arrows), show also correlated expression of HAS2, whereas ZEB1-positive stroma cells (open arrowheads) are either HAS2 positive or negative. Scale bars, 50 µm (left panel) and 20 µm (right panel).

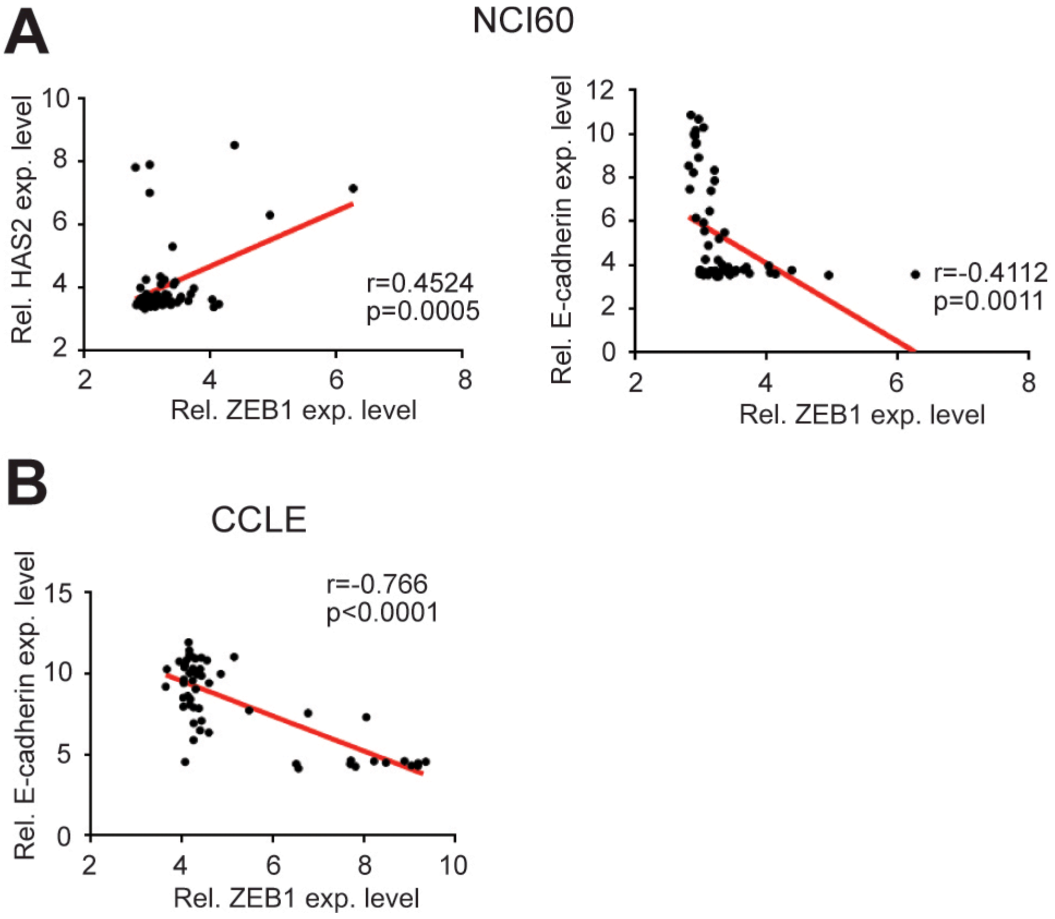

Supplementary Figure S3: ZEB1 and HAS2 are correlated in cell lines of the 'NCI60 panel'. (**A**) Same correlation analysis as in Figure 2 of *ZEB1* and *HAS1*, *HAS2* and *HAS3* in cancer cell lines of the 'NCI60 panel' (GSE5846) showing that only *HAS2* expression is correlated with *ZEB1*. In accordance with *E-cad* repression by ZEB1, *E-cad* and *ZEB1* are inversely correlated. (**B**) A similar inverse correlation of *E-cad* and *ZEB1* is found in breast cancer cell lines of the 'CCLE panel' (GSE36133). Pearson correlation coefficients r and p-values were computed and are indicated.

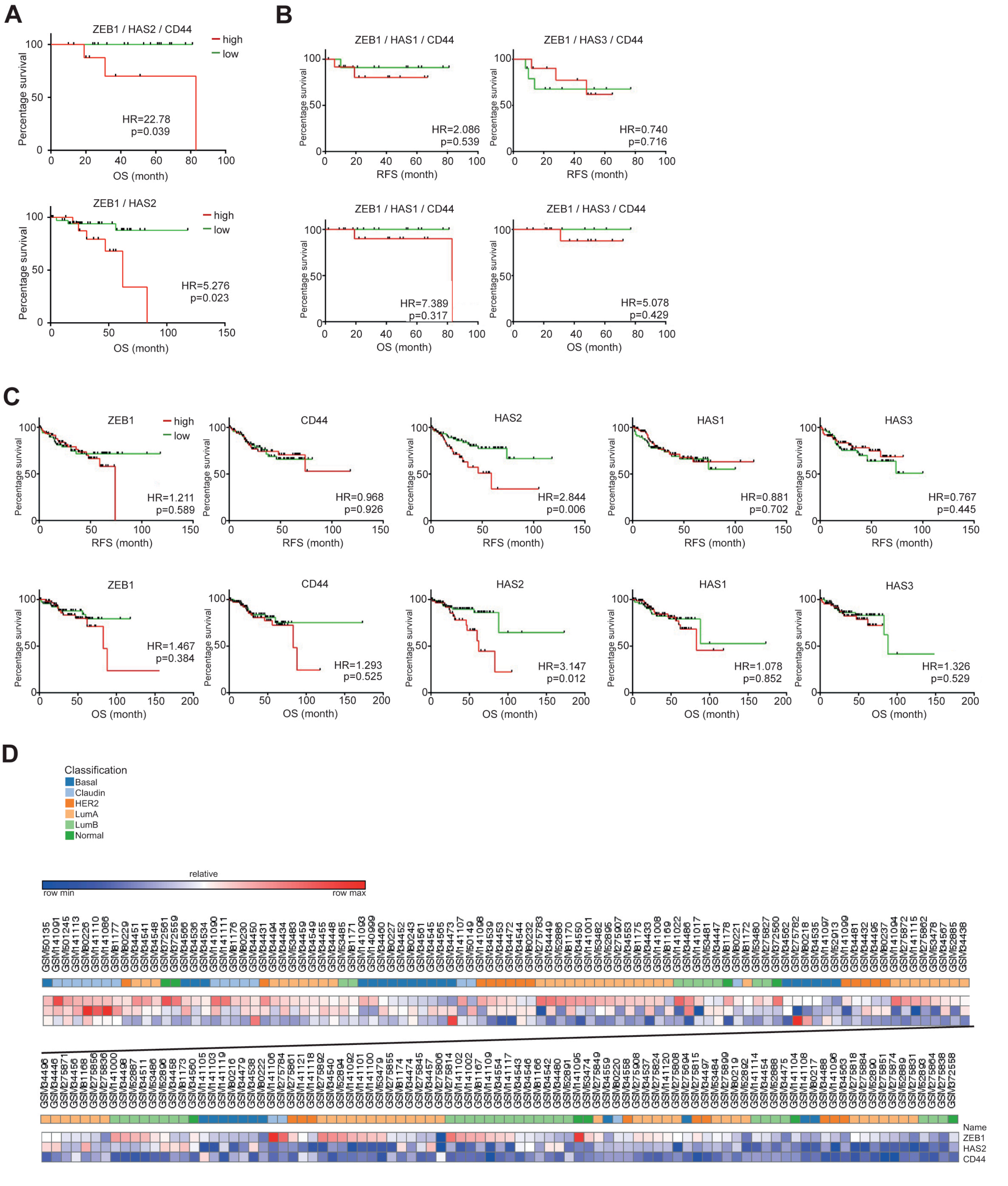

Supplementary Figure S4: *HAS2* expression correlates with early relapse and poor overall survival in breast cancer. (**A**) Expression data of 337 breast cancer patients (GSE18229) were analyzed for relapse-free survival (RFS) and overall survival (OS) in the upper 58%ile and lower 42%ile of the indicated genes, extending Figure 2G. Kaplan-Meier analyses show that high expression of *ZEB1*, *HAS2* and *CD44* and to a lesser extend of *ZEB1* and *HAS2* is correlated with poor OS. (**B**) *HAS1* and *HAS3* in such genesets are not increasing hazard ratios (HR). (**C**) Single gene analysis for survival shows that *HAS2* is correlated with early relapse and poor survival, whereas in this study *HAS1*, *HAS3*, *ZEB1* and *CD44* alone show no correlation to survival. Note, that *ZEB1* levels are also high in stromal cells, explaining a non-significant difference in RFS and OS between high and low level groups. Hazard ratios (HR) and logrank p-values are indicated. (**D**) Complete heat map of all patient samples for expression of *ZEB1*, *HAS2* and *CD44* reveals that the claudin-low subtype is specifically clustered and shows high expression levels of the three genes.

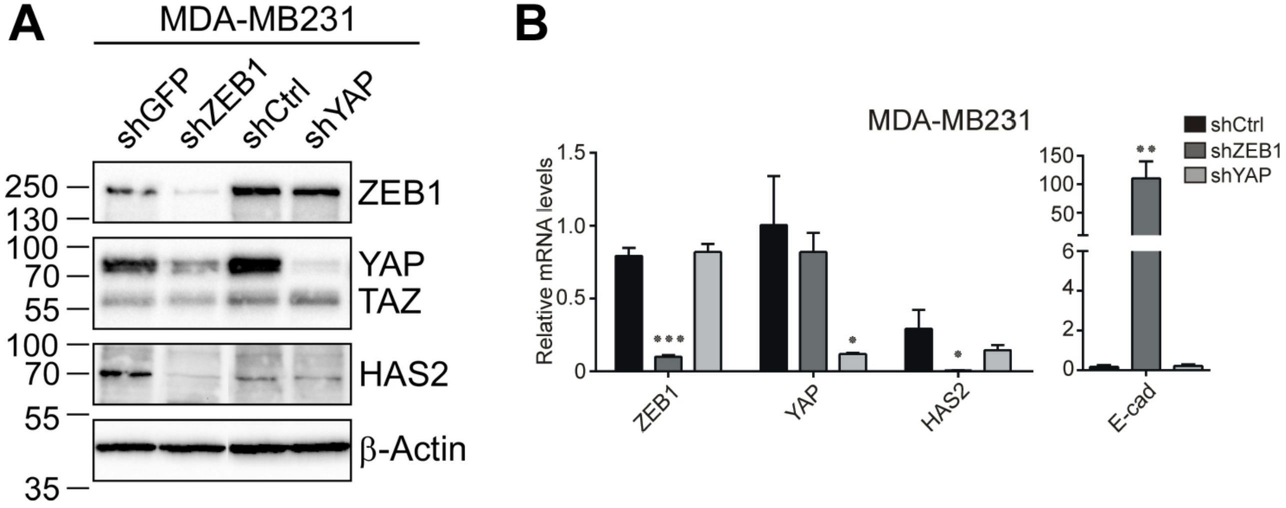

Supplementary Figure S5: HAS2 expression is strongly reduced upon stable knockdown of *ZEB1*. (**A**, **B**) Western blot (A) and qRT-PCR (B) analysis of MDA-MB231 cells with a stable knockdown of either *ZEB1* or *YAP* and their corresponding control cells. Efficient knockdown is confirmed on protein and mRNA levels, whereas TAZ levels are relatively unaffected. In agreement with a transient *ZEB1* knockdown HAS2 is reduced on protein and mRNA levels in shZEB1 cells. Interestingly, HAS2 is also downregulated to 50% upon *YAP* knockdown, although not significantly. Since *HAS2* promoter fragments were not enriched by anti-YAP ChIP this might indicate an indirect regulation. mRNA levels of MDA-MB231 shGFP cells are set to 1.
